# Inflammation accelerating intestinal fibrosis: from mechanism to clinic

**DOI:** 10.1186/s40001-024-01932-2

**Published:** 2024-06-18

**Authors:** Shuzi Xin, Xiaohui Liu, Chengwei He, Han Gao, Boya Wang, Rongxuan Hua, Lei Gao, Hongwei Shang, Fangling Sun, Jingdong Xu

**Affiliations:** 1 Department of Physiology and Pathophysiology, School of Basic Medical Sciences, Capital Medical University, Beijing, 100069 China; 2 Department of Clinical Laboratory, Aerospace Clinical Medical College, Aerospace Central Hospital, Beijing, 100039 China; 3 Key Laboratory of Carcinogenesis and Translational Research (Ministry of Education/Beijing), Department of Renal Cancer and Melanoma, Peking University Cancer Hospital & Institute, Beijing, 100142 China; 4 Department of Clinical Medicine, School of Basic Medical Sciences, Capital Medical University, Beijing, 100069 China; 5 Department of Intelligent Medical Engineering, School of Biomedical Engineering, Capital Medical University, Beijing, 100069 China; 6 Experimental Center for Morphological Research Platform, Capital Medical University, Beijing, 100069 China; 7 Department of Laboratory Animal Research, Xuan Wu Hospital, Capital Medical University, Beijing, 100053 China

**Keywords:** Fibrosis, Inflammation, Intestine, Cytokine, ECM, Intestinal microflora

## Abstract

Intestinal fibrosis is a prevalent complication of IBD that that can frequently be triggered by prolonged inflammation. Fibrosis in the gut can cause a number of issues, which continue as an ongoing challenge to healthcare systems worldwide. The primary causes of intestinal fibrosis are soluble molecules, G protein-coupled receptors, epithelial-to-mesenchymal or endothelial-to-mesenchymal transition, and the gut microbiota. Fresh perspectives coming from in vivo and in vitro experimental models demonstrate that fibrogenic pathways might be different, at least to some extent, independent of the ones that influence inflammation. Understanding the distinctive procedures of intestinal fibrogenesis should provide a realistic foundation for targeting and blocking specific fibrogenic pathways, estimating the risk of fibrotic consequences, detecting early fibrotic alterations, and eventually allowing therapy development. Here, we first summarize the inflammatory and non-inflammatory components of fibrosis, and then we elaborate on the underlying mechanism associated with multiple cytokines in fibrosis, providing the framework for future clinical practice. Following that, we discuss the relationship between modernization and disease, as well as the shortcomings of current studies. We outline fibrosis diagnosis and therapy, as well as our recommendations for the future treatment of intestinal fibrosis. We anticipate that the global review will provides a wealth of fresh knowledge and suggestions for future fibrosis clinical practice.

**Graphical Abstract:**

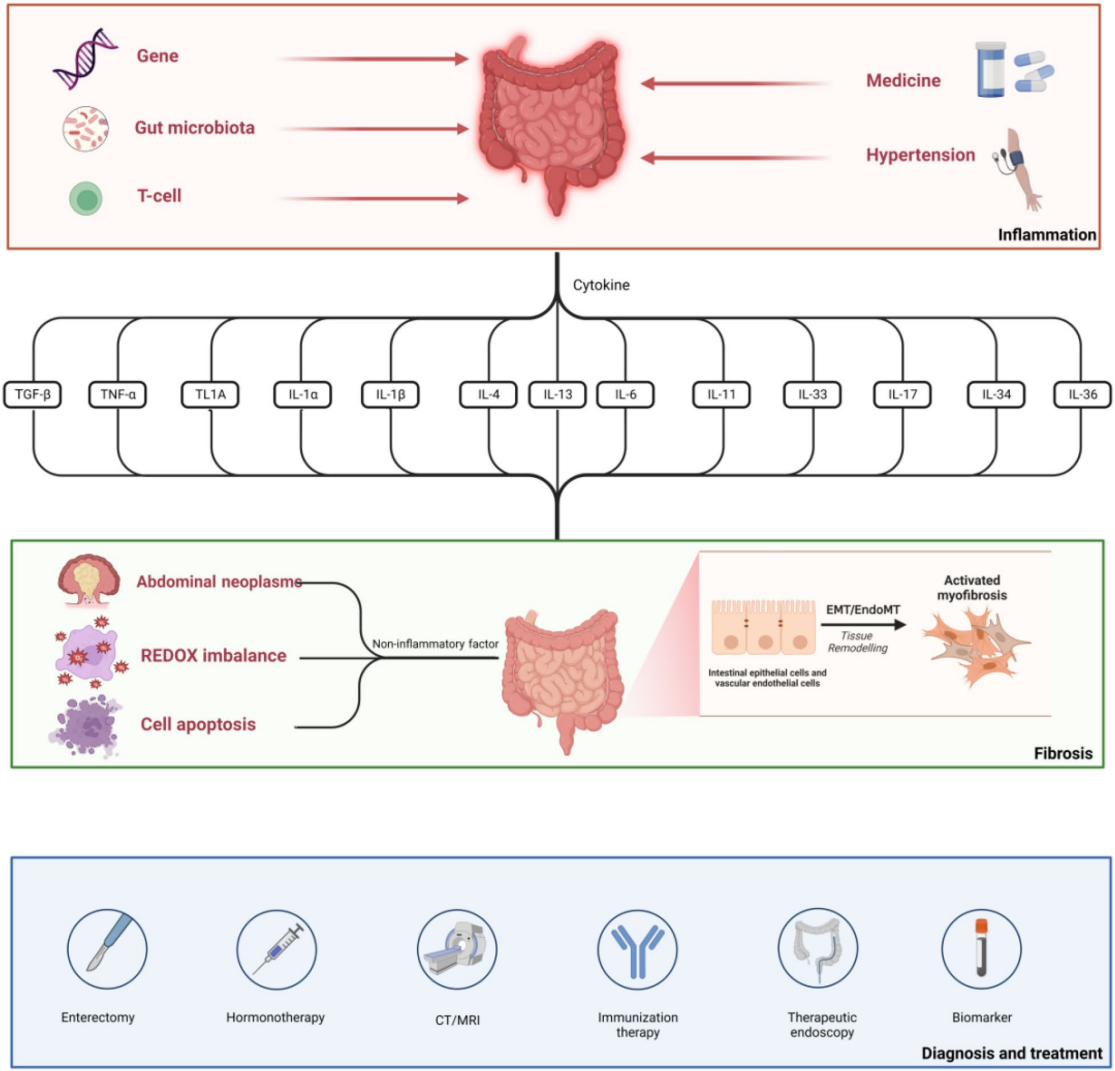

## Highlights

1. Since the intestinal tract is an extremely complex organ of immune regulation, the occurrence and development of intestinal fibrosis are more complex, as well as cardiovascular system, it has received more attention.
2. Fibrosis triggered by prolonged chronic inflammation is an immensely complicated disorder. Considering there is an abundance of inflammatory variables involved, fibrosis therapy becomes challenging
3. Disturbance of the intestinal milieu and cytokines in gut are potential fibrogenesis routes as well.
4. Intestinal fibrosis can substantially impede bodily function, so fibrosis relief is crucial.
5. The bidirectional regulation of cytokines in the cellular route, inflammatory response and fibrosis has attracted wide attention.

## Introduction

Fibrosis can cause harm to any organ and accounts for up to 45% of all fatalities in countries with advanced economies. Fibrogenesis is a physiological, reparative process that might be damaging whenever a hazardous substance persists in the healing process for an extended period of time. Intestinal fibrosis is characterized by excessive deposition of collagens and other extracellular matrix (ECM) components, with gut smooth muscle cells serving as one of the primary mesenchymal cell sources. Fibrosis was long assumed to be a persistently progressing and irreparable process, but preclinical experiments and clinical trials in numerous systems of organs have demonstrated that it is an extremely unpredictable one. It has been viewed as a model of intestinal fibrosis linked with persistent inflammation, as shown in IBD, particularly Crohn's disease (CD). The approach also included an appropriate investigation of cytokine production during the inflammatory phase. Inflammatory bowel disease (IBD) is now widely recognized as a common chronic immune-mediated disease that occurs predominantly in the intestine, and it remains a prevalent ailment putting a substantial strain on the healthcare system. According to the research survey, the morbidity is the highest in North America and Europe, but in recently grown in emerging nations such as Asia, Africa, and South America [1]. Several investigations have corroborated that the pathogenesis of ulcerative colitis (UC) is restricted to the symptoms of chronic persistent inflammation of the colon, and the mucosa and submucosa, whereas CD is more likely to occur in the terminal ileum and ascending colon [2]. Excessive accumulation of ECM accumulation leads to recurrent injury, and hardening of tissues in the process of chronic persistent inflammation, eventually leading to intestinal fibrosis, which is a typical natural outcome of IBD. Fibrosis-related disorders affect more than 30% of CD patients, while UC patients also have moderate fibrosis [3]. Current research has revealed that pulmonary and myocardial fibrosis occurs during the COVID-19 epidemic, although its pathogenesis and anti-fibrosis mechanism remain unknown [4, 5]. The formation of fibrosis is an extremely complex regulatory process involving inflammatory factors and immunological reactions. Other experimental evidence has hinted that hypertension may directly affect digestive tract immunity, thereby affecting the degree of fibrosis, or indirectly regulate sympathetic nerve and intestinal flora, lower intestinal mucosal barrier, and reduce the occurrence of intestinal digestion [6, 7]. Although receiving widespread attention, the anti-inflammatory treatment routinely employed in clinic is mostly ineffectual in the fight against intestinal fibrosis [8]. Thereby, it is critical to explore the mechanism of fibrosis and identify the mediator involved in the process. We summarize the latest evidence on inflammatory factors and immunological response in fibrosis. Simultaneously, we also emphasize the relationship between fibrosis and inflammatory variables, as well as non-inflammatory factors and products, in order to elucidate the research progress, which provides a novel approach for future clinical practice.

## Current viewpoint in the pathogenesis of IBD

Although the etiology of IBD remains largely unknown, it involves a complex interaction between genetic, environmental, and microbial factors [9]. Currently, the pathogenesis of IBD is multifactorial and involves the presence of pathogenic factors, are making significant advancements in figuring out the molecular etiology of IBD [10].

### Microbiota and IBD

The gut microbiota is a complex ecosystem that plays a crucial role in the maintenance of intestinal homeostasis and the regulation of the immune system. Abnormal gut microbiota is one of the pathogenic factors in IBD. Firstly, of the 163 loci discovered, CD occupied 30 loci, UC inhabited 23, and both occupied 110 loci. NOD2, ATG16L1, CARD9, and IL23R were among the genes studied [10]. According to studies on the fibrosis-related genes have depicted that C-X-C motif chemokine ligand 9 (CXCL9), Thrombospondin 2 (THBS2), Matrix Gla protein (MGP), Protein tyrosine phosphatase receptor type C (PTPRC), CD52 molecule (CD52, similar to CD24 molecule CD52), Granzyme A (GZMA, also CTLA3, HFSP), Dermatopontin (DPT), and Decorin (DCN, DSPG2, SLRR1B) may have the function of changing the expression of fibrosis. They are not only significantly expressed in UC and CD, but also in liver and kidney fibrosis [11]. WWOX (WW domain-containing oxidoreductase), on the other hand, is a non-negligible factor in fibrotic stenosis in CD [11].

### Environmental factors

There is no doubt that the variable factors of the environment, such as smoking, diet, drugs, geography, social pressure and psychological disorders are crucial in the etiology of IBD. A study has shown that the deficiency vitamin B can aggravate the occurrence of IBD, but lower the occurrence of intestinal fibrosis [12]. The increased prevalence UC may be associated with a continuous duration of high dosages of nonsteroidal anti-inflammatory agents [10], as well as the environmental pollution [13]. Several hypotheses on the underlying causes of IBD include a dysfunctional immune host response to normal luminal components, infection with a particular pathogen, and/or an inadequate mucosal barrier to luminal antigens.

The etiology of IBD has been linked to dysbiosis or an imbalance in the composition of the gut microbiota. The disturbance of intestinal microenvironment is a potential cause that could not be ignored in the onset of IBD, for example, the diversity of fecal flora in patients with IBD is considerably lowered. Studies have also shown that intestinal adherent invasive Yersiniabactin generated by *Escherichia coli* (AIEC) has been found to induce granuloma and granulomatous colitis in vitro, which is directly linked to enteritis and intestinal fibrosis [14]. At present, there is universal concern that dietary habits and fiber have direct or indirect impacts on the intestinal tract, potentially altering the function of intestinal microbes [15].

### Immune response

Existing evidence implies that inappropriate activation in both acquired immunity and unacquired immunity responses exacerbate intestinal inflammation. Innate immunity generates related immune responses via the recognition of various antigens. Pattern recognition receptors (PRRs) are made up of Toll-like receptors (TLRs) in the membrane and NOD-like receptors (NLRs) in the cytoplasm [16]. Due to its abundant and diversity of T cells, adaptive immunity is more complex link in the specific immune regulation [10]. The immunological abnormalities associated with T helper 1 (Th1) cells are considered to be a major cause of enteritis. IFN-γ derived from Th1 is primary cytokines that mediates the onset of UC and CD, particularly their modulation of immune function as well as targeting of epithelial cells impacted by disease have attracted the most attention. IL-12 also effectively promotes Th2 production with IL-4 as well IL-5/13 [17]. These evidence imply that cytokines interact strongly, exhibiting reciprocal regulation, and even antagonism.

### Hypertension accompanied by intestinal fibrosis

**Fig. 1:**
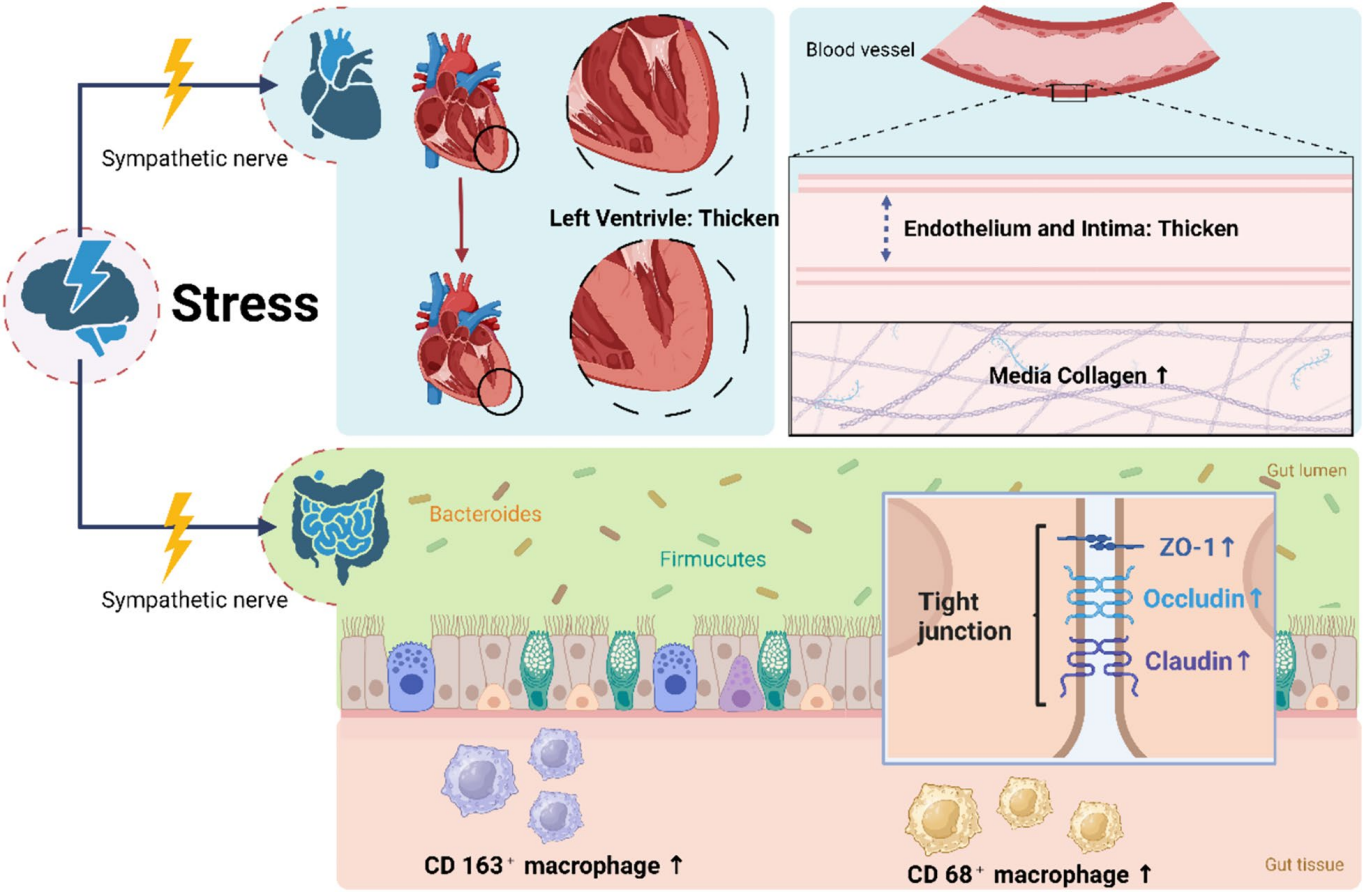
Pathophysiological alterations of cardiovascular and intestinal tract in humans during stress. The pressure signals sensed by the paraventricular nucleus of the hypothalamus go via the sympathetic nervous to thicken the endothelium and intima, increase collagen in the media, and thicken the left ventricle. The expression of occludin, connexin, and ZO-1 was found to be increased, as well as the of CD68^+^ and CD163^+^ Mφs in the intestine

At present, it is generally accepted that hypertension is the leading cause of cardiovascular disease [18], upon growing understanding that there is a probable tight association between hypertension and the pathological alterations of intestinal inflammation. The gut is innervated by peripheral and autonomic nerves, as well as a complex microbial flora that influences the intestinal immune response and mucosal barrier under the intricate regulation of blood pressure [7]. First of all, the tension disorder of hypertension plays an extremely potential role in the onset of hypertension [19]. The hypothalamic paraventricular nucleus detects hypertension signals, conducts them to hypothalamus and regulates neurohormones within the digestive system [7], which may result in higher permeability of the intestines along with lowered tight junctions. Secondly, autonomic nerves can alter intestinal permeability, inflammatory status, and microecological environment in enteritis [7, 20] with the precise process given in Fig. 1. Notably, several researches have corroborated the use of RAS system inhibitors in spontaneously hypertensive rats (SHRs) to verify their antihypertensive, as well as anti-inflammatory and antifibrotic effects. Candesartan, an angiotensin type 2 receptor blocker, has been demonstrated to prevent ileal and colonic lesions in rats as well as enhance intestinal expression of tight junction protein coding genes such as cingulate protein, occludin, and tight junction protein 1(TJP1). Surprisingly, candesartan may alleviate the intestinal mucosal barrier damage based on an increase in serum LPS-binding protein levels [21]. Furthermore, ACEI inhibitors can ameliorate the elevation of SHR blood pressure via retarding the sympathetic nerves tonicity, thereby reversing intestinal dysfunction [7]. Nevertheless, some evidence has indicated that the improvement of intestinal tract by inhibiting renin–angiotensin–aldosterone system (RAAS) is mainly related to intestinal epithelial apoptosis and Th17-mediated immune response rather than directly related to the regulation of blood pressure [22]. So, determining whether the pathogenic alterations in the digestive tract are primarily caused by hypertension, sympathetic nerve activity produced by hypertension signals, or both is challenging. Lastly, hypertension leads to a decrease in the abundance, diversity, and evenness of gut microbes, as well as an increase in the Firmicutes/Bacteroidetes ratio (F/B) [23], which is linked to the decline in acetate and butyrate, along with the cytokine alternation. There is currently a scarcity of research on hypertension-related enteritis. As a consequence of the foregoing experimental findings, we can definitely infer that alleviating the progress of hypertension and sympathetic tone performs a vital part in the occurrence of enteritis as well as gastrointestinal fibrosis and the underlying mechanism is illustrated in Fig. 1.

## Unraveling the underlying mechanisms of fibrosis formation

Given that emergence of fibrosis in the intestinal tract is an elaborate procedure involving multiple elements and mechanisms, we are going to concentrate on the course of fibrosis formation and its underlying mechanism of cytokine shifting within this section.

### The fibrotic process of intestinal inflammation

Fibrosis in IBD has a profound impact on clinical outcomes, including motor abnormalities, anal dysfunction, rectal urgency, and urinary incontinence [24]. Acute inflammation is a key driver of multi-organ fibrosis. The use of bleomycin-induced pulmonary fibrosis and carbon tetrachloride-induced hepatic fibrosis models demonstrated that short-term drug exposure induced epithelial cell apoptosis and hepatocyte necrosis. The aforementioned processes stimulated inflammatory triggers and wound healing responses, which in turn led to the deposition of ECM in the tissues and the generation of fibrosis [25]. In a study on the establishment of a mouse model of enteritis, dextran sulfate (DSS)-mediated epithelial damage and epithelial repair were produced in acute colitis. Following a prolonged dosing cycle, chronic non-self-limiting colitis resulted in intestinal fibrosis due to the combined effects of sustained injury and physiological repair. Chronic 2,4,6-trinitrobenzene sulfonic acid (TNBS)-mediated colitis is characterized by the persistent development of lamina propria fibrosis [26]. A review of the literature indicates that submucosal fibrosis and thickening of the mucosal muscular layer are associated with chronic persistent injury, rather than active inflammation. This indicates that fibrosis and mucosal muscular layer thickening are common complications of progressive ulcerative colitis. The innate and adaptive immune response facilitates the proliferation and differentiation of myofibroblasts, which is of significant benefit for the generation of ECM, a central step in fibrosis. Indeed, the chronic process of tissue repair resulting from a persistent inflammatory response is a key factor in the development of fibrosis. Conversely, the direct removal of inflammatory factors is the most effective method of interrupting the tissue remodeling process and facilitating the gradual transformation of the affected tissue into normal tissue. This is because the dissipation of inflammatory factors leads to the suppression of the continuously increasing ECM and promotes matrix metalloproteinase (MMP)-induced fibrous matrix degradation. Furthermore, it has been demonstrated that the reduction of inflammation is associated with an increase in the deposition of fibrous matrix and a decrease in the degradation of MMPs, resulting in the accumulation of ECM [27]. The fibrotic process described above is the result of a multifactorial combination of factors, including the activation of the coagulation cascade and the combined effects of chemokines and cytokines released by platelets and injured endothelial cells [28]. Four distinct subpopulations of fibroblasts have been identified in the UC, including an activated fibroblast population expressing interleukin (IL)- 33, lysyl oxidase, TNFSF14, and fibroblast reticulocyte-related genes [29]. A full-thickness single-cell RNA atlas report of stenotic ulcerative colitis revealed the heterogeneity of fibroblasts in stenotic CD. The majority of transcriptional changes were observed in the mucosal and submucosal layers of stenotic CD, with fewer changes observed in the lamina propria compared to controls. In particular, specific fibroblast populations, such as CXCL14^+^ and MMP/WNT5A^+^ fibroblasts, exhibited either increased numbers or enhanced transcriptional activity in stenotic CD compared with non-stenotic but inflamed and non-inflamed tissue from the same patients [30]. Nevertheless, clinical observations have indicated that chronic intestinal inflammation does not invariably result in intestinal fibrosis. Celiac disease and lymphocytic enteritis do not appear to be associated with the development of fibrosis or stenosis [31]. This diagram illustrates the various mechanisms by which different inflammatory conditions progress to recovery or fibrosis. In the case of a common clinical condition such as intestinal inflammation, it is of great importance to explore the specific mechanisms that occur and thus provide targets for clinical treatment.

During acute intestinal inflammation, fibrogenesis is a necessary physiological process that assists in tissue regeneration and wound healing. Inflammatory cells release cytokines and growth factors, which stimulate mesenchymal cells including fibroblasts and myofibroblasts to create ECM components such as collagen [32]. This short-lived ECM deposition serves as a provisional scaffold for tissue healing and aids in the restoration of intestinal wall integrity [9]. Once the acute inflammation has subsided, MMPs destroy the extra ECM, and the tissue recovers to its normal structure [32].

Chronic inflammation, such as IBD, causes dysregulated fibrogenesis and excess ECM deposition, culminating in pathological intestinal fibrosis. Pro-fibrotic cytokines such as TGF-β, IL-13 and other soluble mediators activate mesenchymal cells over a sustained period [33]. The contribution of additional cell types, such as epithelial cells (EMT), endothelial cells (EndoMT), and bone marrow-derived fibrocytes. Impaired ECM breakdown caused by an imbalance of MMPs and their inhibitors (TIMPs). The fibrotic process becomes self-perpetuating and inflammation-independent, resulting in excessive ECM build-up, intestinal wall thickening, and strictures development [34]. Other variables that promote fibrosis include the gut microbiota, TLRs, and plasminogen activator inhibitor-1 (PAI-1) [35].

In conclusion, whereas fibrogenesis is a natural reparative process during acute inflammation, dysregulated and persistent fibrogenesis caused by chronic inflammation eventually results in pathological intestinal fibrosis in conditions such as IBD.

### Potential mechanisms for the development of intestinal fibrosis

**Fig. 2:**
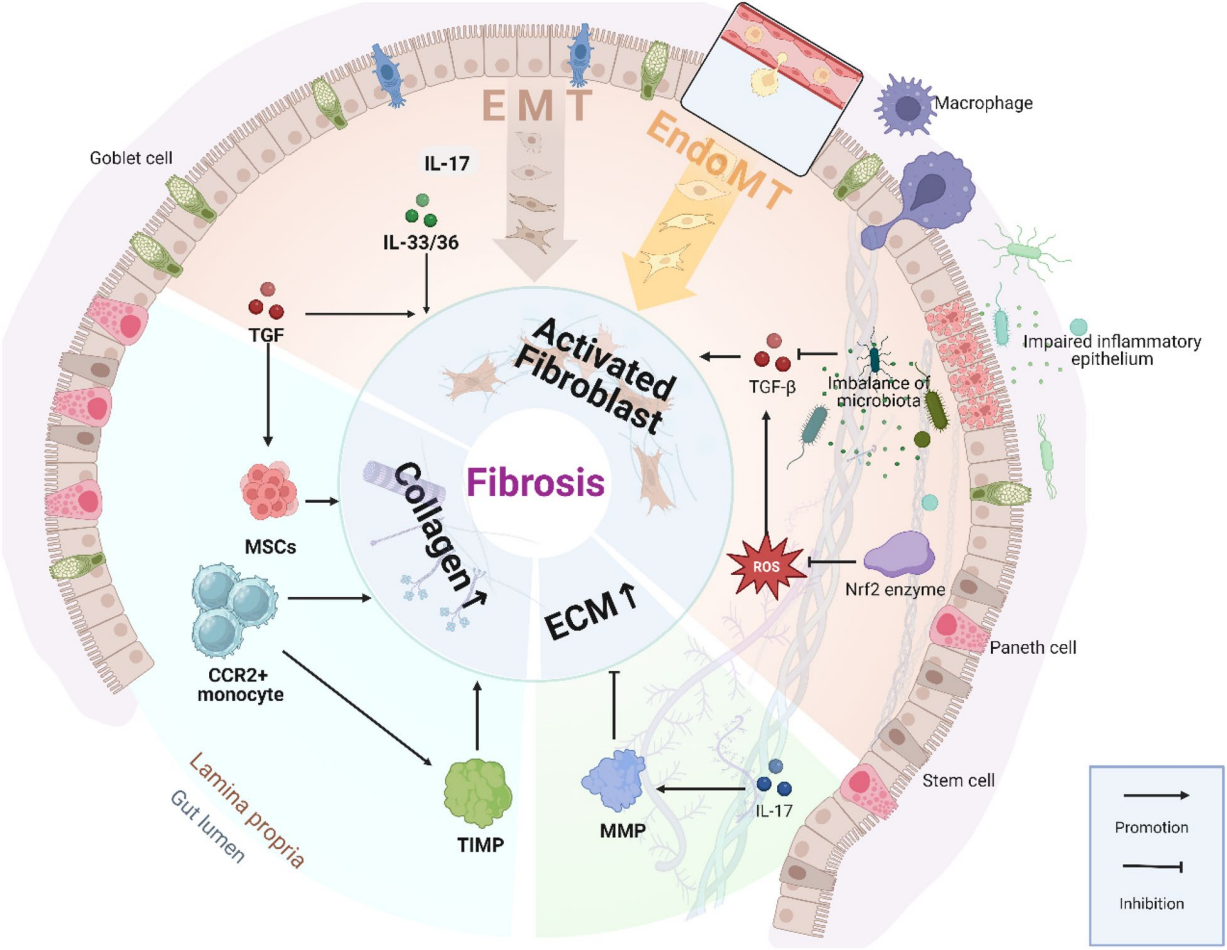
Pathophysiological diagram of intestinal fibrosis. Inflammation is triggered by distinctive inflammatory and associated non-immune cells, as well as microbiota that enhance epithelial and endothelial EMT and EndoMT and catalyze myoblast activation through pathways such as cytokines, growth factors, and oxidative stress. This process ultimately results in the formation of fibrosis

Fibrosis is described as abnormal accumulation of ECM and ongoing accumulation of collagen, resulting in compromised cell integrity and healing. Numerous studies have revealed that chronic enteritis with IBD patients, particularly CD, leads to repeated and sustained epithelial cell damage, as well as long-term immune response characterized by tissue overgrowth, sclerosis and scarring to cope with the damage, which eventually leads to chronic progressive fibrosis [2, 24, 36]. ECM components such as collagen and fibronectin can also be secreted by intestinal mesenchymal cells, albeit in tiny amounts. The amount of mesenchymal cells of diverse sources aggrandizes locally throughout the course of fibrosis induced by multiple factors [31]. The most important elements influencing fibrosis include inflammatory substances from several sources, such as transforming growth factor (TGF), tumor necrosis factor (TNF), growth factors, as well as different interleukins. These mediators generally promote the fibroblast activity, as well as proactive differentiation and dedifferentiation of mesenchymal cells. This type of fibroblast activity results in three related cell phenotypes: fibroblasts, subepithelial myoblasts, and smooth muscle cells, all of which can lead to proliferation both fibroblasts and myofibroblasts [31, 37]. When the stability of MMP and TIMP is disrupted, the colonic wall undergoes morphological remodeling. The former are proteolytic enzymes capable of cleaving ECM components, whereas the latter counteracts their hydrolytic activity and maintains the two enzymes' balanced homeostasis [38]. Fibroblasts and myofibroblasts are transformed from epithelial and endothelial cells via the EMT or EndoMT processes to repair intestinal mucosal barrier abnormalities and restore tissue integrity up until recovery, which is another mechanism depicted in Fig. 2. EMT, on the other hand, is the process through which epithelial cells convert into fibroblasts and take on their functions. Similarly, several of the aforementioned proinflammatory factors can promote fibrosis by stimulating EMT and EndoMT [36]. Current research in fibrosis involves filling information gaps, which might improve understanding of the fibrosis mechanism. Although inflammation is essential for the onset of fibrosis, it does not always impact the progress of fibrosis due to the additional variables associated with the data processor.

### Multiple factors affect intestinal fibrosis

In addition to EMT and EndoMT, intestinal fibrosis is most likely to be linked to intestinal microecology, oxidation–reduction (REDOX) imbalance, and intestinal cell apoptosis [39]. As depicted in Fig. 2, IBD fibroblasts have been revealed to be directly modulated by the microflora [40], for example, *Streptococcus* and *lactobacillus* were shown to be prevalent in ileum fibrosis, while sulfite-reducing bacteria were found to be negatively associated with ileal fibrosis [39]. TGF-β is elevated in IBD fibrotic tissues due to an imbalance in gut flora. Antibiotic medication or outright suppression of the TGF-β signaling cascade can avoid fibrosis caused by excessive ECM accumulation [41]. Despite ongoing study, the underlying mechanism by which intestinal flora promotes the progression of intestinal fibrosis remains elusive. Oxidative stress is defined as an imbalance in oxidation and antioxidation that modifies the equilibrium of reactive oxygen species (ROS) / reactive nitrogen species (RNS) and, consequently, weakens the intestine's oxidative defense of the system [39]. In the colon of IBD patients, researchers uncovered increased oxidants and reduced Nrf2 enzyme activity, indicating that oxidative stress contributes to fibrosis. When exposed to ROS, and Nrf2 products may eliminate ROS, hence lowering oxidative stress [42]. Scavenging ROS can suppress TGF-β1-mediated EMT, illustrating the diverse function of oxidative stress in modulating inflammation-related fibrosis across several signaling pathways. Multiple studies have demonstrated that the BCL2 family proteins, which have been shown to stabilize mitochondrial membranes and prolong cell life, can improve fibrogenesis by improving fibroblast survival and facilitating fibroblast differentiation [39, 43]. The content of α-SMA and COL1A1 were considerably lowered in fibroblasts treated with a modest dosage of BCL2 antagonist in a control set of assays, corroborating the results presented above [43]. In practical, fibrosis remains a challenging to cure. The high prevalence of inflammation in patients' bodies facilitates to generate fibrosis, which is a major impediment to patient rehabilitation [2]. Despite the absence of a full understanding of non-inflammatory fibrosis, ongoing research into the underlying processes can considerably advance the targeted therapy for intestinal fibrosis.

## Cytokines serve as a crucial bridge between the immune response and fibrosis

The digestive tract is the body's most potent immune system. Due to long-term exposure to food antigens and complex and diverse microbial flora, the intestinal tract not only needs to protect the integrity of its mucosa and barrier to maintain daily digestion and absorption function, but also needs to maintain the state of "inflammation preparation" to respond to the invasion of various antigens. When there is inflammation, the intestinal microenvironment becomes extremely complicated, and many different types of cells release cytokines, such as interleukin (IL) and TNF, which can trigger inflammatory response via the action of complicated cellular subnets and accelerate, postpone, or even reverse the process of intestinal fibrosis. However, it is unresolved how this process is generated by the combined activity of various parts or the mutual influence of different components [44]. Therefore, in this section, we will further provide extend insight into the role of cytokines involved in fibrosis and summarize the pertinent signaling pathways in Table 1 in order to propose novel recommendation for future clinical practice.

### TGF-β

TGF-β is ubiquitous, adaptable, and essential for survival. They are essential for growth and development, as well as inflammation and repair. The role of TGF-β in homeostatic and pathological processes hints that it could potentially be beneficial for both the detection and management of inflammatory and fibrotic ailments.

#### General biological characteristics of TGF-β

TGF-β has long been considered as the essential mediator of intestinal inflammation and fibrosis, as well as a primary contributor to enteritis fibrosis [45].TGF-β1/2/3, bone morphogenetic protein, and several growth- and differentiation-promoting factors belong to the TGF-β family [46]. TGF-1 receptor 1 (TR1) is transmembrane receptor with serine/threonine kinase activity, as well as TR2 [47]. TGF-β1 pathway malfunction or overexpression can cause gastrointestinal inflammatory, fibrotic hyperplasia, and cancer cell mutation [48]. The ongoing expansion of the ECM is one of the reasons for the constant high production of TGF-β1 in sick organs [49].

#### TGF-β involved in the proliferation of multiple types of cells

Cell surface serine/threonine kinases and Smad family effector predominantly hinder TGF-βdownstream proliferation. TGF-β promotes carcinogens in the late stages of tumor formation by suppressing the immune system and altering the epithelial tumor cell differentiation, thereby known as EMT [50]. Malfunction of the TGF-β cascade can lead to tumor development. GDF10 is muted due to promoter hypermethylation, which may operate as a tumor suppressor gene in NPC via TGF-β/Smad/NF-κB signaling pathways [51]. TGF-β restricts the majority of cells, including upper skin cells and endothelial cells. IGFBP-3 and TGF-β are antagonized by the V-type TGF-β receptor (t-β-V). The reason why cancer cells are able to escape TGF-β regulation and become more aggressive may be related to TGF-β inhibition of epithelial and endothelial cells [52]. TGF-β limits different cell proliferation, notably fibroblasts. Cyclosporine promotes gingival fibroblast proliferation via the TGF-β and Sonic hedgehog (Shh) pathways [53]. According to a substantial body of research, TGF-β1 has a powerful inhibitory impact on hepatocytes proliferation either in vivo or in vitro study as well as in normal, regenerative, neoplastic, and preneoplastic liver nonparenchymal cells. Fausto's group evaluated high-affinity binding sites for TGF-β1 binding to rat hepatocyte membranes and discover that the mRNA transcription of TGF-β1 in patients with persistent hepatic disorders was closely matched to the expression of pro-fibrotic proteins or nucleic acids [54]. TGF-β receptor signaling may also be required for the pancreas to prevent β-cell death following proliferation, and the TGF-β pathway is beneficial to the regulation of β-cell homeostasis [55]. Inhibition of TGF-β signaling, for another, extends the β-cell lineage. Especially in the classical TGF-β signaling pathway, Smad3 inhibits the secretion of islet β cells to maintain the homeostasis of insulin in the body [56]. TGF-β1 inhibits keratinocyte proliferation by inhibiting c-myc transcription, acts as a chemotactic factor for fibroblasts, has indirect mitogen for certain mesenchymal cells, and induces ECM deposition [57, 58]. Similarly, TGF-β1 pathway also has an impact on reproductive system proliferation, as well as to uterine epithelial cells and prostatic duct cells [59, 60]. Through the TGF-β signaling route pathway, zoledronic acid can suppress fibroblasts proliferation and migration [61, 62]. TGF-β possesses two type I receptors, ALK-1 and ALK-5. Due to current research, the properties of TGF-β/ALK5 pathway and TGF-β/ALK1 pathway are diametrically opposite, the former has an inhibitory effect on stimulating endothelial cell proliferation and metastasis, while the latter has a promoting effect [63]. In another investigation, ALK-5 has been shown to be involved in growth stimulation, and the novel ALK4/5/7 kinase inhibitor SB-431542 reduced TGF-β induced growth activation, as well as Gleevec and AG1296 [64]. TGF-β1 might promote the MSCs proliferation while hindering the osteogenic differentiation, which may be related to the Smad3-dependent swift nuclear translocation of β-catenin in MSCS [65].In particular, TGF-β1, has a multidimensional effect in promoting tumor progression, which is a tumor suppressor in its initial phases of tumor and becomes a carcinogenic factor in the terminal phases. When the PDGF-B gene is not methylated, TGF-β/Smad signaling can induce the expression of PDGF-B and promote the proliferation of glioma [66]. TGF-β inducing cytostasis is demonstrated in many cell types, but it may also influence proliferation, apoptosis, dormancy, and autophagy in others. This dual role of TGF-β is vital in the future therapeutic practice, and it additionally implies a bright future for cancer patients.

#### TGF-β fibrosis-related downstream pathway expression and influencing factors

**Fig. 3:**
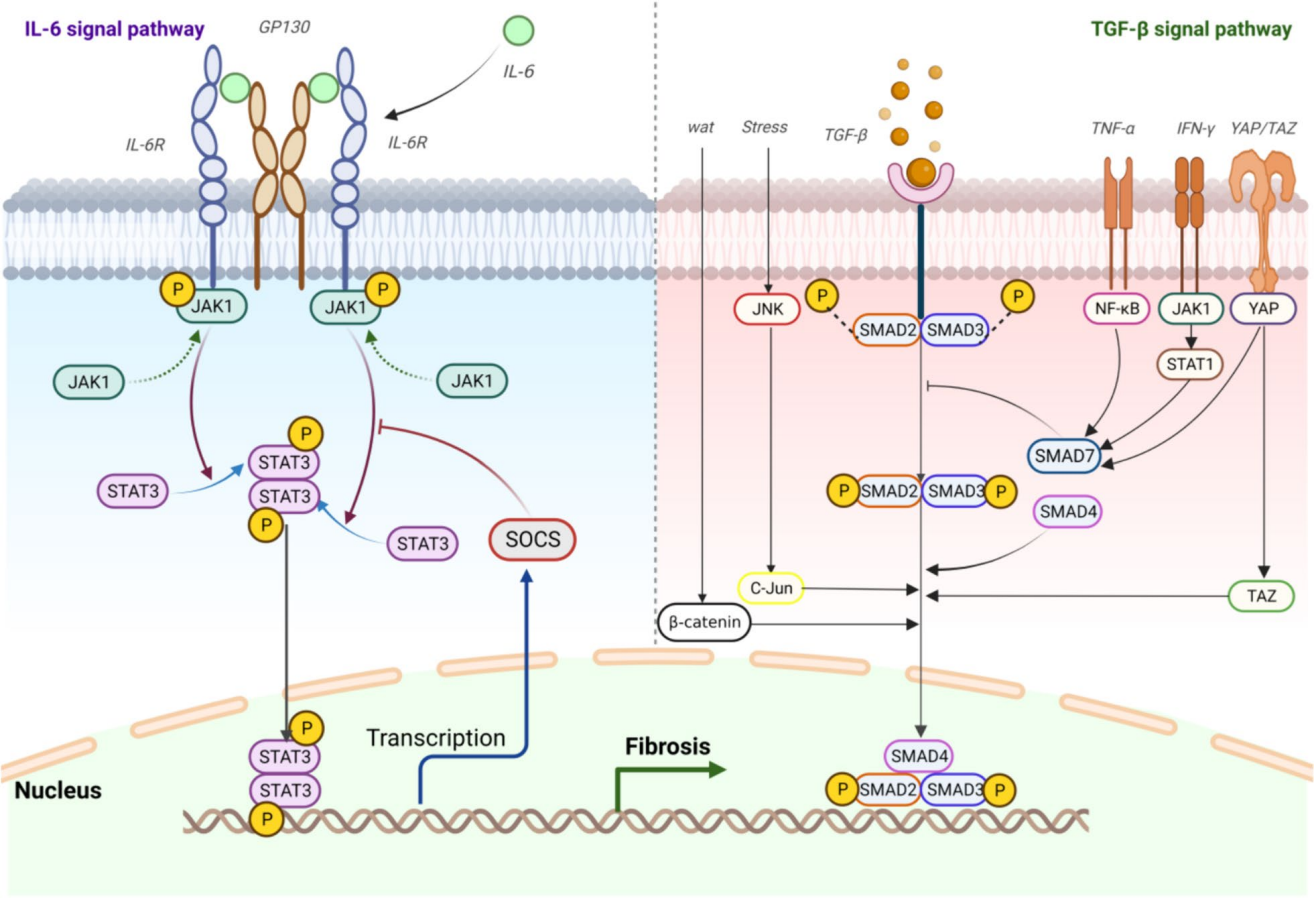
Diagram illustrating putative IL-6 and TGF-β signaling processes involved in intestinal fibrogenesis and interaction with other signaling outlets. The processes behind the TGF-β and IL-6 signaling pathway in fibrosis depicted schematically

Aside from what has already mentioned above, the Smad protein is the primary intracellular effector of TGF-β1, which can activate Smad for signaling, thus raising the expression of pro-fibrotic genes. Numerous investigations have revealed that aberrant TGF-β1/Smad transduction is the primary cause of fibrosis. Among them, TGF-β1 receptor complex phosphorylates Smad2/3/4 then coupled with phosphorylated products, and the complex may then primarily control and express target genes, which is finished in the nucleus [49]. Meng’s Team explored the function of Smad4 in regulating TGF-β signaling in mice with unilateral ureteral obstruction and noticed that Smad4 activates Smad3, which leads to the activation of downstream promoters and promotes the co-ligation of Smad3 with the COL1A2 promoter, confirming the Smad3 and Smad4 play a promoting role in liver fibrosis [67]. Tsuchida K's group revealed that deleting smad4 in mesangial cells suppresses TGF-β1-induced ECM deposition, supporting the fibrotic crucial role of Smad3 and Smad4 [68]. Smad2 and Smad7, on the other hand, possess a protective role in fiber development. Smad3 inhibition caused lower level of type I collagen and blocked epithelial–myofibroblast transition as well as Smad2. Furthermore, research on the influence of Silibinin on the expression of Col I revealed that inhibiting the Smad2/3-dependent signaling pathway alleviated Col I upregulation [69], as well as Smad4 via enhancing Smad3 responsiveness and promoter activity. An experiment employing doxycycline induction in a mink lung epithelial (Mv1Lu) cell line demonstrated that Smad7, a negative feedback regulator, decreased activation of TGF-β1/Smad signaling, more particularly, Smad7 inhibited PAI-1 as TGF-β target genes [70]. As depicted in Fig. 3, Smad7, which has a negative feedback regulatory function, would protect against TGF-β1-mediated fibrosis [49, 71]. Smad6 antagonizes the Smad pathway activated by bone morphogenetic protein (BMP) type I receptors ALK-3 and ALK-6, whereas Smad7 inhibited Smad signaling via TGF-β and BMP [72]. In various disease models, TGF-β1 or its downstream signaling pathways can be suppressed to slow the advancement of renal fibrosis, whereas the overexpression of TGF-β1 accelerates the progression. According to the data shown above, we have reason to believe that TGF-β1 signaling pathways, both Smad-based and non-Smad-based, can cause fibrosis and be linked to myofibroblast activation, ECM formation, and degradation of ECM [45]. TGF-β, phosphorylated Smad2-Smad3 (PSMad2-3), and TIMP-1 expression were considerably greater in the stenotic area than in the non-stenotic area. TGF-β transcripts were greater and the PSMad2-3 response to TGF-β was stronger in stenotic intestinal mucosa myofibroblasts than in non-stenotic locations. These evidence revealed that TGF-β pathway inhibition promotes MMP-12 myofibroblast formation and migration [73]. In one research, Smad7 antisense oligonucleotide treatment alleviated colitis along with reduced collagen deposition and stopped fibrosis in mice, which was associated with lower TGF-β1 production and p-Smad3 protein expression [74]. With the rapid development of biological technology, the role of vaccines has attracted wide attention.TGF-1 vaccination has been proven in tests to lower excessive TGF-1 bioactivity, which may aid in the prevention and treatment of intestinal fibrosis [75]. TGF-β1 caused a dose-dependent pro-fibrotic response in organoids. According to immunofluorescence evidence, spironolactone reduces the occurrence of intestinal fibrosis by inhibiting the migration of colonic myofibroblasts [76], which is a crucial element in wound healing [77]. Furthermore, intestinal microecological circumstances might improve immunological homeostasis by modulating TGF-β production [41]. Inhibition of AXL signaling, for example, blocks TGF-β1, thereby relieving the fibrotic process of myofibroblasts in the colon [78]. GRP78 protein overexpression can activate TGF-β1 signaling and induce fibrosis [79]. Nrf2 inhibits enteritis and fibrosis via negatively regulating the ROS/TGF-1/SMADs pathway [42], FnE can catalyze the growth and differentiation of fibroblasts and influence the development of myofibroblasts via activating TGF-β. It is particularly noteworthy that integrins is indispensable in this process [80]. The role of TGF-β in the pathogenesis of fibrosis has also been documented in some clinical trial reports. The antifibrotic drug colchicine was found to significantly reduce TGF-β expression [81]. Tissue nonspecific alkaline phosphatase (TNAP) has been identified as a potential anticardiac fibrosis drug, exhibiting the capacity to exert an inhibitory effect on fibrosis by inhibiting the AMPK-TGF-β1/Smads pathway [82]. In the context of patients with systemic sclerosis, transforming growth factor beta (TGF-β) induces the expression of DNA methyltransferase 3A (DNMT3A) and DNMT1 in fibroblasts in a SMAD-dependent manner. This results in the repression of cytokine signaling inhibitory factor 3 (SOCS3) expression via promoter hypermethylation. The downregulation of SOCS3 promotes the activation of STAT3, which in turn facilitates the transformation of fibroblasts to myofibroblasts and fibrogenesis in vitro and in vivo. The downregulation of SOCS3 facilitated the activation of STAT3, which, in vitro and in vivo, promoted the transformation of fibroblasts into myofibroblasts, collagen release, and fibrosis [83]. From the above evidence, the regulation of TGF-β on fibrosis is an extremely complex process, in which many related signaling pathways are involved.

### TNF-α

At early stages of intestinal inflammation, the proinflammatory effect of TNF-α is more potent, albeit its exact mechanism is unknown [84]. TNF-α induces tissue inhibition of metalloproteinase-1 (TIMP-1) expression while limiting matrix metalloproteinase-2 activity and collagen degradation with activating ERK1/2. TNF-α may, to different degrees, promote the secretion of IL-8, McP-1, MMP-1 and MMP-2 [85]. In particular, TNF-α also cause inflammatory and fibrotic via the IRAK–NFκB pathway. It influences affects chemokine secretion and ECM metabolism accumulation of IL-1β [85]. TNF-α has a fibrotic impact mainly completed through the encoding TNFR2 gene, which drives fibroblast proliferation via ERK1/2 and inhibits collagen degradation via TIMP-1 generated through STAT3 [84]. TNF-α reduced mRNA expression of SATB2 and MUC5B in intestinal epithelial cells and goblet cells, also promoted mucosal damage and junction disintegration in the human generated pluripotent embryonic stem cell-driven gastrointestinal organoids (HIO) model [86]. Furthermore, TNF-α can also stimulate colonic myofibroblast migration [87]. In a rat model of IBD, anti-TNF-α antibodies reduced intestinal inflammatory symptoms and fibrosis [88]. Similarly, various investigations have been carried out to lower TNF-α for anti-inflammatory and antifibrotic in patient purposes. Nonetheless, the mechanisms are still being addressed [89]. TNF-α-induced epithelium damage can be mitigated by probiotics, which is an interesting phenomena to consider [90]. Some individuals who received anti-TNF-α therapy early had a lower incidence of intestinal stenosis [91]. In the infliximab trial, it was found that anti-TNF-α medications might stimulate TIMP-1production and further improve the migration capacity of myofibroblasts in CD, resulting in the reduction of MMP activity and wound healing [92]. Clinical trials have demonstrated that rifaximin-α administration to patients for 30 consecutive days results in a significant reduction in peripheral blood TNF-α expression, while simultaneously promoting the growth of gut microorganisms enriched in TNF-α and IL-17, with the aim of reducing systemic inflammation, particularly in the gut, and preventing fibrosis [93]. Based on the evidence presented above, we may infer that the anti-TNF-α therapy has substantially ameliorated the incidence of stenosis and fibrosis.

### TL1A

Tumor necrosis factor like cytokine 1A (TL1A), as a part of the TNF superfamily of members (TNFSF15) [94], is a vita cytokine that modulates the positioning and inflammatory process of intestinal epithelial inflammation, which eventually leads to fibrous stenosis [95]. Dominant TL1A expression directly induced the production of the TNFSF15 haplotype but also enhance the risk of small intestinal stenosis [96]. TL1A attaches to the death domain receptor 3 (DR3) and performs as a costimulatory of IFN-γ secretion [18], and this relationship may be detected in a range of cells with diverse degrees of differentiation, including cells of the immune system, epithelial cells, and fibroblasts. In the context of immunological reactions, endothelial responses to IL-1β and TNFα can promote TL1A production, which is also produced in Mφs and dendritic cells that conduct signals to recognize TLR [97]. The amount of TL1A-positive cells, mRNA and proteins in inflammatory cells are all altered by inflammation and the pathological proceedings of CD. The proliferation of DR3^+^T cells in the lamina propria of intestinal mucosa was correspondingly increased [18]. In inflamed intestinal mucosa, TL1A may operate on a large number of DR3-expressing lymphocytes, such as CD4^+^ and CD161^+^ lymphocytes, and eventually activate IFN-γ and IL-17 pathways [98]. In patients with IBD, increased expression of the E-cadherin was associated with the decreased TL1A expression, whereas the opposite was observed for the mesenchymal markers (FSP1 and α-SMA). TL1A-induced EMT may be related to TGF-β1/Smad3, which leads to the increased expression of IL-13 and EMT-related transcription molecules (ZEB1, Snail1). Simultaneously, anti-TL1A antibody or BMP-7 might both alter TL1A-induced EMT [16]. Other investigations have identified a relationship between TGF-β1 and Smad3. In a chronic colitis T cell transfer model, anti-TL1A antibodies inhibit intestinal fibroblast activation and reduce collagen synthesis, resulting in anti-inflammatory and antifibrotic effects [94]. Preclinical research suggest that targeting the TL1A pathway may be an effective therapeutic method for intestinal fibrosis in IBD, such as CD. A phase 2a clinical trial (TUSCANY) demonstrated that an anti-TL1A antibody PF-06480605 minimized fibrosis gene expression in patients with ulcerative colitis (UC), including genes involving extracellular matrix remodeling and fibrosis [99]. However, there are also research results that are inconsistent with the above conclusions. Overexpression of TL1A causes fibrosis that is reliant on certain microbial populations [97]. Modulation of TL1A-DR3 can independently influence fibrosis [96, 100, 101].

### IL-1

Seven of members within the IL-1 group either proinflammatory properties (IL-1α, IL-1β, IL-18, IL-33, IL-36α, IL-36β and IL-36γ), while the additional four are antagonistic (IL-1RA, IL-36RA, IL-38) or anti-inflammatory properties (IL-37) [102].

#### IL-1α significantly mediates the inflammatory response of fibroblasts

IL-1α not only modulates inflammatory process, but it also initiates traditional the classic cytokine activity via membrane receptors and impacts the transcription and translation program of genes [102]. Different cell types involving epithelial cells, Mφs, monocytes, and endothelial cells, elevate or release IL-1α in response to diverse factors [102], although IL-1 dominates innate immunity, it may also be involved in acquired immunity. All IL-1 receptors possess the Toll-IL-1 receptor (TIR) domain. IL-1α can exacerbate intestinal inflammation by inducing cytokine production in mesenchymal cells. In the interaction between intestinal epithelial cells (IEC) with fibroblasts, IL-1α may be involved in the amplification and maintenance of inflammation [103]. TLRs, on the other hand, promote inflammation via bacteria, microbial products, viruses, nucleic acids, and closely related damage-associated molecular patterns (DAMPs) [104]. The costimulation of TLR3 ligands in the presence of IL-1α in medium derived from injured lung epithelial cells greatly improves the inflammatory phenotype of primary human lung fibroblasts (PHLFs). This evidence depicts that IL-1α is particularly crucial in eliciting proinflammatory responses in fibroblasts, and this impact is heightened in the presence of double-stranded RNA [105]. Bleomycin resistance resulted in neutropenia and decreased collagen deposition in IL-1-deficient model [105]. This provides essential insight into how inflammation may cause fibrosis in a variety of tissues, nevertheless, there are numerous routes involved in inflammation and fibrosis, making its participation and management a highly difficult subject.

#### Indirect role of the IL-1β pathway in fibrosis generation

IL-1 is not ordinarily produced in healthy cells, instead, it is usually secreted and acted upon by substances generated by damaged cells or pathogenic bacteria, as well as TLRs and other pattern recognition receptors (PRRs) that are activated to cause protein accumulation in cells [102]. IL-1r1 /MyD88 is a typical inflammatory pathway downstream of the IL-1R family [106]. Fluindione, as an anti-inflammatory agent, has no direct impact on IL-1β expression but exerts two effects. Firstly, it inhibits NLRP3 inflammasome and down-regulates caspase-1, lowering the level of IL-1β pro-cleavage into IL-1β [107]. Furthermore, the IL-1β/IL-1R1/MyD88/NF-κB pathway was blocked, and IL-1β was suppressed in a dependent manner [108]. What is more noteworthy is that recent studies corroborate that IL-1β can induce the generation and secretion of the pro-fibrotic factor osteopontin (OPN) and upregulate the release of downstream factors like as IL-6 [109]. IL-1β enhanced the production of type I and type IV collagen while boosting IL-8, McP-1, MMP-1 and MMP-2 secretion [85]. There is a large number of experiments to prove that the increasing the expression of IL-1β can cause myofibroblast accumulation, and extracellular accumulation of collagen and fibronectin [110], or even cause fibrosis in many organs, such as heart [111], liver [112, 113], kidney [114], lung [108, 115]. In clinical studies, IL-1β was identified as a pivotal cytokine that sensitizes Th17 cells to IL-12 and effectively induces the differentiation of cells that produce proinflammatory factors, including IL-17, IFN-γ, and granulocyte–macrophage colony-stimulating factor (GM-CSF) [116]. Despite preclinical research suggest that IL-1α/β have an involvement in intestinal fibrosis, there is insufficient information on the current state of clinical trials assessing IL-1 inhibition as a treatment for the condition [117].

### IL-4 and IL-13

Cytokines IL-4 and IL-13 have a plenty of overlapping physiological and immunoregulatory impacts on B cell population [118]. The varieties of IL-4 receptors have been uncovered in recent years. One is composed of IL-4Rα and γc chains, and the other is constructed up both IL-4Rα and IL-13R1. Both of which could simultaneously react with IL-4 and IL-13, marking a new breakthrough in the research of IL-4 and IL-13 receptor complex, which contributes to fibrotic progression in various tissues. IL-4, produced by T lymphocytes and mast cells, accumulates ECM by specifically raising the steady-state levels of type I and III procollagen and fibronectin mRNA. Then, fibroblasts were activated to produce more type I collagen transcripts [119]. According to several research, the mRNA expression of collagen type I, III, and IV increased in human liver fibroblasts treated with IL-4 [120]. Inhibiting IL-4 in oxazolone-induced colonic inflammation may significantly alleviate symptoms of intestinal fibrosis [121]. IL-13 is a cytokine that is largely involved in the progression of fibrosis becoming more widely acknowledged [122]. There is evidence to suggest that IL-13 has a positive effect on collagen deposition in intestinal smooth muscle of CD patients. The receiving effect of IL-13Rα2 is indispensable for the positive activation of TGF-β1 promoter by IL-13. The mechanism by which IL-13 promotes fibrosis in UC involves the direct induction of collagen expression and the activation of TGF-β1 promoter through IL-13Rα2 signaling. Disparities in the impacts of IL-13 on inflammation and fibrosis have been documented. IL-13 promotes collagen accumulation within the muscle microenvironment of Crohn's disease. In CD, IL-13 induces tissue remodeling and fibrosis. The IL-13 signal transduction pathway and IL-13Rα2 are driving factors in the comprehensive colon fibrosis. Collagen synthesis in myofibroblasts during fibrosis is derived from TGF-β1 roused, which promotes the downstream release of IGF-I and EGR-1[123, 124]. IL-13 transcript expression was higher in was greater in persons with CD fibrosis. CD activates the IL-13 pathway, which can create IL-13 by infiltrating IL-13Rα1^+^ and KIR^+^ innate lymphoid cell population, restrict MMP synthesis in fibroblasts, and lead to matrix breakdown and excessive collagen deposition [125]. Preclinical models of fibrotic disease indicate that the activity of IL-13 on multiple cell types, including Mφs and fibroblasts, is associated with the initiation and perpetuation of pathological fibrosis. In controlled experiments of clinical treatment, the expression levels of IL-13, IL-4, IL-13Rα2, and IL-13-induced target genes were found to be significantly higher in lung tissues of idiopathic pulmonary fibrosis compared with controls. This suggests that IL-4 and IL-13 may exert a fibrotic effect at the genetic level [126]. Currently, the status of clinical trials exploring TL1A inhibition for intestinal fibrosis is ambiguous. Preclinical testing indicates that IL-4 and IL-13 enhance fibrosis in a variety of organs, including the lungs and gut. Several clinical trials have evaluated IL-4/IL-13 antagonists in IPF patients, with inconsistent results. The search results do not provide any definitive details on the current state of clinical studies testing these cytokines as therapeutic targets for intestinal fibrosis in IBS [127]. Overall, the evidence suggests that targeting IL-13 may be an intriguing approach for the treating UC fibrosis.

## Box 1 Comparing the similarities and differences pathways activated by IL-4/IL-13 in fibrosis

Both IL-4 is vital fibrosis mediators in many tissues, as well as IL-13. IL-4/IL-13 signaling promotes megakaryocyte proliferation and transforming growth factor surface expression, which contributes to the fibrotic development of myeloproliferative neoplasms (MF). IL-13 has been demonstrated in certain studies to induce fibrosis by boosting autocrine CTGF signaling in fibroblasts and inducing the pro-fibrotic transcription factor Snail, although IL-4 has not been proven to activate similar pathways. In numerous animal models of pulmonary fibrosis, IL-13 is considered to be more pro-fibrotic than IL-4.

### Intersectionality of the functions of IL-6 and IL-11 in the progress of fibrosis

IL-6 and IL-11 are two related cytokines that share some similarities but also have unique functions. The IL-6 signaling has been categorized into three types including classic signaling, trans-signaling, and cluster signaling [128]. The conventional IL-6 signaling pathway encompasses the identification of IL-6 by IL-6 receptor α (IL-6Rα) element and glycoprotein 130 (gp130) signal transduction subunit. This type of signaling is primarily limited to hepatocytes, Mφs, neutrophils, and resting T cells, as these cells express membrane-bound IL-6Rα, control inflammation and immunity [129, 130]. IL-6 activates the JAK/STAT and MAPK cascades, which preserve mucosal integrity via the gp130, LIF, and OSM receptors [128, 131] as more details depicted in Fig. 3. In response to IL-1β or LPS stimulation, intestinal glia in the mouse gut released more of IL-6 [128], which has been proven in studies to aggravate inflammation in two ways: directly driving the proliferation and differentiation of lymphocytes, and indirectly/directly catalyzing inflammation via the nervous system. Until now, its anti-inflammatory and mediating properties, notably in the mucosal barrier, have increasingly been understood [128]. Multiple IL-6 targeting therapeutic strategies are being exploited, mostly notably cancer, inflammation, and fibrosis [132]. Compared to IL-6, IL-11 plays a more substantial role in advancing gastrointestinal carcinogenesis via IL-11/STAT3 signaling, making IL-11 signaling a viable therapeutic target for treating [133].

Under physiological settings, myofibroblasts and injured epithelial cells release IL-11, a pro-fibrotic cytokine, but under pathological conditions, smooth muscle cells (SMCs) IL-11R express enhancement [134]. IL-11 activates colonic fibroblasts and epithelial cells via phosphorylating STAT3. Fibroblast markers and genes related to cell proliferation and tissue healing were clearly visible in IL-11-positive cells. This evidence suggests that IL-11 might serve as a precursor route in both the fibrotic and tumor microenvironments [135]. Intestinal inflammation, fibrosis, and intestinal wall thickening were seen in IL-11^+^ SMC mice, as well as ERK and STAT3 activation [134]. IL-11 has been shown in studies to cause fibrosis in certain lung cancer cells, mostly through enhancing cell motility, invasion, and EMT. This is an essential function in pathological tissue fibrosis and tumor development [136]. Moreover, the article reported both IL-6 and IL-11 may promote adipocyte proliferation in cystic ovary syndrome (PCOS) rats by activating AKT/STAT3 signaling pathway [137]. Although the reason behind it is unresolved, it provides novel challenge for dealing with the obesity-related disorders in the clinical practice. A clinical study has identified IL-6-mediated collagen-induced signaling pathways. The trans-signaling of IL-6 is dependent on STAT3 and indirectly enhances signaling from TGF-β and the downstream mediator Smad3 [138]. In the treatment of idiopathic pulmonary fibrosis, it was demonstrated that protease-mediated lysis of lung Mφs is a crucial step in the production of sIL-6Rα (sIL-6Rα). In vivo neutralization of sIL-6Rα resulted in attenuation of pulmonary fibrosis, as evidenced by reductions in lung myofibroblasts, fibronectin, and collagen. In vitro activation of IL-6 trans-signaling has been demonstrated to promote fibroblast proliferation and extracellular matrix protein production, effects that are associated with the progression of pulmonary fibrosis [139]. A phase 2 trial of the anti-IL-6 antibody PF-04236921 or IL-6 pathway blockage in Crohn's disease patients showed reduced disease activity. Nevertheless, the investigation was discontinued due to possible hazards of gastrointestinal perforations [140]. Clinical research clearly indicates IL-11 is a significant cause of fibrosis in various organs, including the gut, via activating stromal cells [141]. The search results show no current or completed clinical studies explicitly testing IL-11 inhibition for intestinal fibrosis. Overall, IL-6 and IL-11 have the function of regulating immune response, inflammation, fibrosis, and tissue integrity. While they share some similarities in their activities, they also have diverse roles in different tissues and physiological contexts.

## Box 2 Cytokines from the IL-6 family

The IL-6 family of cytokines, which includes IL-6, IL-11, ciliary neurotrophic factor (CNTF), leukemia inhibitory factor (LIF), oncostatin M (OSM), myocardial trophin-1 (CT-1), cardiotrophin-like cytokine (CLC), and IL-27, is an ensemble of cytokines with an analogous structure and communication route. The IL-6 family of cytokines govern body metabolism, handle the hepatic acute phase reaction, promote B cells, and influences the connection between T cells and effector T cells [142]. Assuming cognate receptors are present, all members of the IL-6 family possess a four-helix bundle structure known as a hexamer or a tetramer, depending on the cytokine concentration and the specific receptor subunits are able to recognize and connect gp130.

## Box 3 Cluster signaling in IL-6 signaling

Cluster signaling, also known as IL-6 transduction, is a type of signaling that occurs often between cells. Cluster signaling in dendritic cells and T cells has recently been discovered in order to connect the gp130 receptor complex, collaborate with IL-6/IL-6Rα complex, undertake conduction through associated pathways, and eventually modify the immune-related diseases. IL-6 signaling dysregulation has been linked to rheumatic arthritis, multiple sclerosis, IBD, and psoriasis.

### IL-33

IL-33 is particular type of cytokine belonging to the IL-1 category. Its structure is based on the β-trefoil structure of molecules. It is encoded by the *IL-33* gene and is expressed in numerous different types of cells. In many of these cells, IL-33 has been demonstrated to be a nuclear and cytoplasmic in a variety of these cells, indicating that it may be released from these cells. IL-33 is composed of 270 residues and possesses a conserved N-terminal nuclear domain and a C-terminal IL-1-like cytokine domain. ST2 signals are interlinked with IL-33 primarily via ST2 orphan IL-1 receptor recognition ligands, with which the cross-action was investigated utilizing characterization analysis to simulate the IL-33/ST2 complex in solution.

Multiple different types of cells generate IL-33, particularly epithelial cells, endothelial cells, and myofibroblast. Being thought to be an alarm protein, IL-33 is promptly generated and binds to its corresponding ST2 receptor, inducing and propagating type 2 unacquired immune response and allergy-related disease [143, 144]. Evidence in vivo experiments reveal that the tissue-derived immune cells, such as mast cells, group 2 innate lymphoid cells (ILC2), and regulatory T cells (Tregs) [144], express the ST2 receptor [145]. The IL-33/ST2 axis exerts anti-inflammatory and anti-fibrotic properties in multiple organs. It impacts the fibrosis development in two parallel ways (macrophage-dependent mode and independent mode), contributes to the hepatic fibrosis and pancreatitis, and mediates renal epithelial injury and EMT. Blocking the IL-33/ST2 signaling axis has been postulated as a potential treatment method, however clinical data are currently limited. TLR4 signaling regulates pro-fibrotic cytokines such as TNF-α and IL-12p40 in Mφs and myofibroblasts, perhaps contributing to intestinal fibrosis [146]. Furthermore, IL-33 is an early indication of activated fibroblasts and myofibroblast trans-differentiation in ulcers [147]. In the heart, IL-33 can directly limit the inflammatory response of proinflammatory Mφs, postponing chronic rejection and fibrosis [148, 149]. IL-33 has been identified as an attainable pro-fibrotic cytokine, with studies demonstrating its role in inducing intestinal fibrosis [36]. In the inflammatory gut, pericryptal fibroblasts generated substantially greater amounts of IL-33. The axis may cause stem cells in gut epithelial precursor cells to undergo differentiation into Paneth cells, goblet cells, and enteroendocrine cells, as well as higher mucin production. To prevent tissue damage and intestinal mucosal integrity, IL-33 activates ILC2 to generate bidirectional regulatory protein (AREG), which subsequently interacts to epidermal growth factor receptor (EGFR) [150]. In rats with dextran sulfate (DSS)-induced damage to the gastrointestinal tract, IL-33 can activate ILC2 cells to alleviate intestinal inflammation while also modulating genes to remodel crypt morphology [150]. Notably, proinflammatory microorganisms were found in greater level in IL-33-/-mice, as well as IgA deficits, which leads to chronic colitis-associated bacterial survival [151]. AIEC induces intestinal fibrosis by adjusting ST2 expression in intestinal epithelial cells via flagellin [152]. Infliximab therapy substantially raised IL-33synthesis but lowered mRNA within the colon mucosal of the active UC individual [150]. Much of the preceding information implies that IL-33 plays a crucial part in the reconstruct epithelial cell integrity, reduces inflammation, and has a certain correlation with fibrosis.

### Novel hints of the diverse capacities of IL-17 in fibrosis

In humans, IL-17 is produced via CD4 Th17 and CD8 Tc17 cells known as T helper 17 cells upon disulfide-linked homodimers being subjected to upstream activation with IL-23 and comprises IL-17A/B/C/D/E/F. It can promote the release of CSFs through downstream reactions and finally complete the mobilization of mature neutrophils in bone marrow. The earliest member of the IL-17 family, IL-17A, is a critical effector in the fight against viral infections, inflammatory lesions, autoimmune disorders, and cancer [153]. IL-17 has been linked to organ fibrosis via boosting the fibroblasts proliferation and IL-1 in endothelial cells of the vascular system. In vitro studies show that IL-17 and IL-17-producing cells participate in the pathophysiology of the disease in hepatic fibrosis by sustaining inflammation and promoting fibroblast proliferation in collaboration with IL-1β. Considering increased expression of IL-17 has been observed in the intestinal mucosa of patients with IBD, IL-17 may facilitate the progression of IBD, which is mainly related to intestinal mucosal immune response, for using neutralizing antibody, IBD symptoms were effectively relieved. Furthermore, IL-17A dominates all immune responses and occurs in the gut, including CD, whose intestinal mucosa samples were found to have high levels of EMT and IL-17A but lower level of E-cadherin in EC-6 cells. Induction of IL-17A, led to the hypersecretion of vimentin, snail, and α-SMA [133]. The serum and intestinal IL-17 secretion were enhanced in mice with TNBS-induced intestinal fibrosis and TL1A has been found to have an unexplored mechanism of inflammatory driven fibrosis, which may be connected with local activation of downstream IL-17A expression in IBD patients. Meanwhile, anti-IL-17 antibody therapy substantially attenuated intestinal fibrosis, as demonstrated by reductions in the transcription and translation levels of various proteins, such as collagen III, TNF-α, TIMP-1, and MMP-2 [154, 155]. These results imply that the fibrotic process of CD is mainly regulated by IL-17A rather than IL-17E. Clinically, IBD, as well as patients with CD who have been treated with anti-IL-17A or anti-IL-17RA neutralizing antibodies, did not decrease but increased. Current evidence is insufficient to support the effect of IL-17A or IL-17RA antagonism in CD, even if CD is found to be alleviated by anti-IL-23P40 and p19 monoclonal antibodies. A research discovered typical poor migration and excessive collagen and TIMP-1production in mucosal isolated subepithelial myoblasts (SEMFs) of CD patients in HSP47, finding consistent with IL-17A receptor expression of SEMF. This implies the immobilization of IL-17A myofibroblasts and the degradation of ECM protein by stimulating MMP-3. Similar findings were obtained with CCD-18CO cells. Accordingly, inhibiting of HSP47 had a deleterious influence IL-17A-induced collagen I secretion [156]. It was proven that Il-17a-neutralizing wild-type mice and IL-17A-knockout mice had negative effects on bleomycin-induced fibrosis and collagen deposition, confirming the characteristics of IL-17-mediated inflammation and fibrosis. Another study, however, revealed that bleomycin and paraquat-mediated pulmonary fibrosis is not IL-17 reliant. In a mouse model of colitis-associated fibrosis, treatment with anti-IL-17 antibodies reduced fibrosis markers like collagen III, TIMP-1, and MMP-2, as well as pro-fibrotic cytokines IL-1β, TGF-β1, and TNF-α [157]. In the early stages of HIV infection, there is a rapid and severe depletion of intestinal CD4^+^ T cells. This results in the impairment of the mucosal barrier and the subsequent onset of chronic systemic inflammation. The administration of panobinostat was observed to result in a decrease in the frequency of CD69^+^ T cells in the intestinal lamina propria and an increase in IL-17A mRNA expression in intestinal epithelial cells. This indicates a potential role for IL-17 in the ongoing repair of the intestinal barrier and its involvement in the process of fibrosis [158].

In vitro studies have shown that IL-17A is pro-fibrotic during CD and influences myofibroblasts’ ability to produce collagen and TIMP-1 as well as their migration, upon which have demonstrated that IL-17A directly interacts with colonic myofibroblasts, and it is a crucial initiating element for stricture development during CD [159], all of which are mediated by IL-17A/ IL-17RA axis [160]. However, a recent study found that IL-17A can be isolated from the induction of myofibroblasts independent of inflammation in IBD living tissue in vitro. Notably, Clinical trials of the drug found that anti-IL-17 therapy worsened the enteritis and prognosis of IBD and was discontinued. If fecal calprotectin (FC) is elevated, clinical evaluation is required to confirm surgery or rediagnosis of enteritis. Once active IBD is diagnosed, IL-17 should be disabled immediately, whereas in patients with quiescent IBD, alternative treatments should be preferred [160].

### IL-34

IL-34 in vertebrates is a secreted dimer glycoprotein that is more conserved than CSF-1 in mammals and birds. IL-34 became known through an in-depth proteome study in 2008 and was initially identified as a related factor capable of generating multiple tissues and controlling monocytes and Mφs via the CSF-1 receptor [161]. IL-34 and M-CSF-1, despite the low homology in amino acid sequences but remarkably comparable tertiary structure, can bind in the overlapping region of MCSF-1r. Due to their hydrophilic/hydrophobic interaction with the receptor MCSF-1r, they may activate multidimensionally and respond to different biological activity signals in diverse forms by affecting two distinct receptor, such as protein-tyrosine phosphatase zeta (PTP-ζ) and CD138 (Syndecan-1) [161, 162]. Due to the fact that IL-34 frequently replicates as mRNA in the body, it can render one vulnerable to disease [163].

Considering IL-34 is implicated in an array of pathological processes ranging from inflammation to cancer, its concentration has been revealed to rise during fibrosis development and might be employed as a particular fibrosis diagnostic marker. IL-34 has been found to be to be a novel modulator of human and exploratory IBD and upregulated or even to sustain inflammatory pathways in the gut, especially in lamina propria monocytes (LPMCs) taken from a typical colon by TNF-α and toll-like receptor ligands, but to be decreased after treatment with infliximab. IL-34, on the other hand, was predominantly up in the jejunum and ileum, whereas M-CSF1 was increased in the ileum and cecum [164]. The information presented above suggests that IL-34 has a variety impact regulated in the development of enteritis by gene molecules. TNF promotes the release of IL-34 in undivided LPMCS isolated from normal colon samples from normal muscle tissue, whereas blockade of TNF with TNF-neutralizing antibodies reduced IL-34 synthesis [165]. Through p38 MAP kinase-dependent process IL-34 encouraged fibroblasts to express COL1A1 and COL3A1, as well as produce collagen in ECM cells. IL-34, which is abundant in CD, promotes the formation of intestinal collagen fibers. In celiac disease mucosal samples, the inflammatory pathological condition caused by IL-34 and M-CSFR-1 was shown to be more severe than that of CD. Lower collagen synthesis and wound healing have been associated with IL-34 downregulation in CD fibroblasts [166]. IL-34 and M-CSF additionally decrease the generation of collagenase and MMP1, thereby raising the synthesis of collagen [167]. The impact of IL-34 in the pathogenesis of pulmonary fibrosis has been examined in a rat lung injury model, and it came to light that differentiation of M2Mφs was the dominant factor, and it can also induce an increase in the production of pro-fibrotic factors and promote collagen I production in hepatic stellate cells, indicating its involvement in the progression of liver fibrosis [167]. Of particular interest is the fact that IL-34 is closely associated with the active phase of hepatitis and has been found to be an essential indicator of a prediction model of liver fibrosis [168]. According to our learning, IL-34 is implicated in amplifying fibrotic processes across many diseases, yet the search results do not indicate any current or finished clinical trials assessing IL-34 as a therapeutic target for fibrosis. The above evidence further gives fresh proposals regarding the mechanism of action of IL-34 in IBD and other diseases is unclear, revealing its biological diversity.

**Table 1:** Summary of correlation between cytokine signal pathway and fibrosis

Cytokine	Agonist	Antagonist	Pathways involved in fibrosis	Impact on gut fibrosis	Refs.
TGF-β	SRI-011381	Sclerostin、PRDC、Nbl1/Dan CV2 fragment	TGF-β1/Smad	Enhance the expression of pro-fibrotic genes, promote the activation and migration of myofibroblasts, regulate ECM, inhibit the secretion of MMP	[169]
TNF-α	htr-9TNF-p55 (rTNF-p55)	PGRN	ERK1/2、STAT3	Encode TNFR2 gene, inhibit collagen degradation, stimulate colonic myofibroblast migration	[170]
TL1A	IFN-γ, FcγR	C03V	TL1A/DR3	Promote EMT, stimulate fibroblast activation with collagen synthesis	[171]
IL-1α	–	Anakinra/ canakinumab	TGFβ/SMAD activated by IL-13	Potent activator of fibroblasts and a reactivator of intestinal inflammation	[172]
IL-1β	–	Anakinra/ rilonacept	IL-1β/IL-1R1/MyD88/ NF-κB	Stimulate OPN secretion, stimulate secretion of type I / IV collagen, myofibroblast accumulation, fibroblast production, promote MMP secretion	[173]
IL-4	Y124D/S125D	dupilumab	IL-4/IL-13 axis	Increased expression of type I and III procollagen and fibronectin mRNA	[120]
IL-13	–	dupilumab /Lebrikizumab	STAT3	Inhibit MMP secretion	[174]
IL-6	Sil-6/subunit IL6ST	Tocilizumab / sarilumab/satralizumab	JAK/STAT、MAPK	Promote fibrosis, induces the phosphorylation of STAT3, trans-signaling via a complex of IL6-soluble gp130	[175]
IL-11	–	W147A/sIL-11R	ubiquitous gp130 receptor、TGFβ1 STAT3、ERK	Promote EMT	[135]
IL-33	–	IL-33trap RMST2-2	IL-33/ST2	Improve inflammation, repair intestinal structure, promote the expression of tight junction proteins	[176]
IL-17	–	Secukinumab, Ixekizumab, brodalumab	TGF-β1–dependent/ independent/mediating the EMT_	Promote EMT and the expression of HSP47, as well as type I collagen	[133]
IL-34	–	anti-IL-34 antibodies	MAPK, PI3K-Akt, JAK, and NF-κB /Mø activation by CSF-1 and/or IL-34	Promote the expression of COL1A1 and COL3A1 genes and collagen secretion, decrease the expression of collagenase and MMP-1	[167]
IL-36	IL-36α, IL-36β, IL-36γ	IL-36Ra,	MyD88/IRAKs/NF-κB/MAPK	Promote fibrosis, the specific mechanism is unknown	[177]

### IL-36

IL-36 belongs to IL-1 family with proinflammatory properties. The IL-36 binds with matching IL-36 receptor to variable degrees (IL1RL2/IL-1Rrp2/IL-36 receptor dimer). IL-36A, IL-36B, and IL-36G activate the IL-36 receptor, whereas IL36-RA inhibits it. Overexpression of IL-36 in keratinocytes, respiratory epithelium, and various immune cell types has been shown to induce inflammation in both acquired and non-acquired immunity. The physiological and pathological roles of IL-36 are still under investigation. However, some evidence implies that IL-36 may serve as a bridge between inflammation and fibrosis.

#### Intestinal inflammatory fibrosis effect of IL-36

**Fig. 4:**
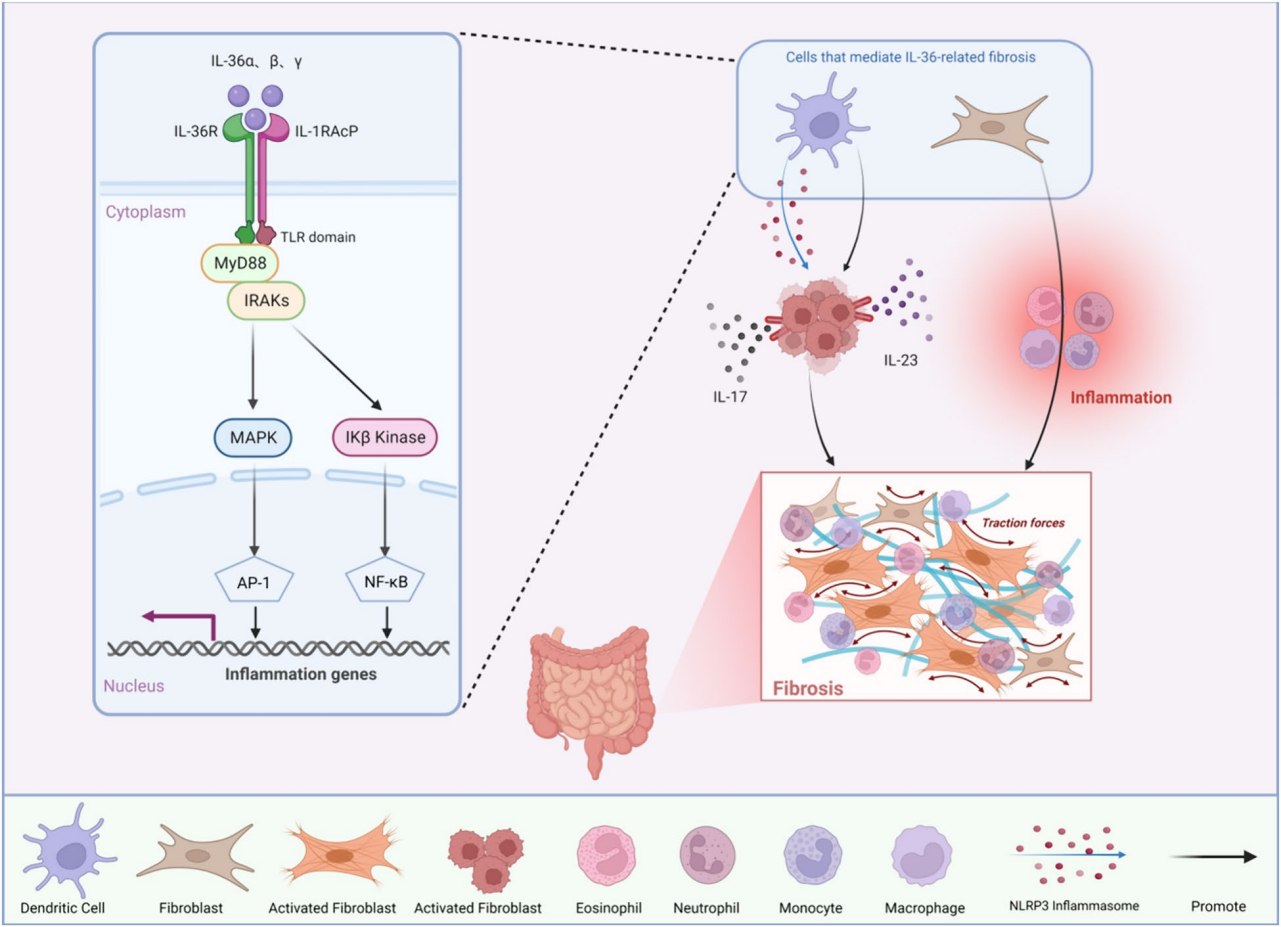
Diagram illustrating the of IL-36 cell signaling pathway in fibrosis process. IL-36 interacts to the IL-36R, which is heterodimerized with IL-1R3 (IL-1RAcP). The IL-36R/IL-36/IL-1R3 complex recruit MyD88 and interleukin-1 receptor-associated kinases (IRAKs), activating the mitogen-activated protein kinase(MAPK) and nuclear factor-κB (NF-κB) pathways. Dendritic cells and fibroblasts are activated and eventually fibrosis occurs. Direct induction shown as a solid black line, indirect induction as dotted black lines

IL-36 triggers downstream proinflammatory pathways by binding to heterodimeric receptor complexes and signaling through intracellular functional domain coding, which has been associated to psoriasis, systemic lupus erythematosus (SLE), rheumatoid arthritis (RA), and IBD. Overactivation of IL-36 is linked to IBD through the classical MyD88–IRAK–TRAF–TAK–TAB pathway, establishing the classical pathway as primary mechanism. MAP kinase and NF-κB pathway are activated, which can promote the inflammatory process of IL-36. The specific mechanism is shown in Fig. 4. Due to several research, the inflammatory substances Th1 cells are a potential driver of IL-36-induced inflammation. The expression of IL-36 receptor antagonist in vivo is down-regulated, which promotes IL-36 action, and the growth of intestinal *A. muciniphila* is related to it. Similar to other factors, IL-36R activates fibroblasts and induces tissue fibrosis through collagen remodeling, which regulates the enzymatic response. Chronic colitis and intestinal fibrosis are ameliorated when the IL36R gene is inhibited or knockout in mic, hinting that inhibition of IL-36R signal may be used as a clinical intervention in patients with IBD [178], which are further confirmed by the evidence of IL-36 isoforms, as well as the IL-36R/IL-1RAcP complex, being upregulated in IBD [179]. Therefore, IL-36 is a crucial cytokine that cannot be ignored in the fibrogenesis process. In the subsequent section we are going to address the function of IL-36 in fibrosis and present our view on how the usage of IL-36 modulates the incidence of fibrositis.

The *IL-36* genes, which include the agonists IL-36α, IL-36β, and IL-36γ, as well as the antagonist IL-36RA, can be identified on human chromosome 2q14.1, and are predominantly expressed in keratinocytes, bronchial epithelium, brain tissue, and monocytes/Mφs, as are the corresponding receptors IL-36R and IL-1RAcP [177]. IL-36 is generated by a wide range of cell types [180]. Elevated quantities of IL-36α and IL-36γ were detected in the intestinal tract of mice as well as mucosal specimens from enteritis patients, which may be associated to tissue damage and intestinal flora activity [181]. To evaluate the involvement of IL-36 in the pathophysiology of enteritis, Russell's team analyzed samples of DSS-induced enteritis from wild-type (WT) and IL-36R ^−/−^ mice to investigate the role of IL-36 in the pathophysiology of enteritis. According to the results presented above, experimental rats missing IL-36R exhibited fewer colitis symptoms and less inflammatory cell infiltration of neutrophils and Mφs [182]. Similarly, several studies have revealed that individuals with IL-36R depletion have a considerable deficit in inflammation alleviation and eventually have more severe colitis with worse mortality and a larger intestinal ecological burden [183–185]. This might imply that inhibiting IL-36R medication may represent a potential therapeutic strategy for IBD fibrosis. Due to its different affinity, IL-36 has a wide variety of effects, including the promotion of pulmonary inflammation and the mediation of synovial inflammation [178, 186].

Thoroughly mapping the expression of IL-36 isoforms throughout time within a given model system, such as IBD or another, in order to identify isoforms that are predominantly present in different stages of disease. Investigating the potential role of IL-36 modulation as a strategy for preventing or treating inflammatory and fibrotic diseases affecting various organs, including the kidney, lung, and intestines. A clinical cross-sectional study involving the examination of intestinal tissues from IBD and non-IBD patients revealed an increase in mRNA expression of IL-36 family members in the colonic mucosa of patients with active ulcerative colitis. Furthermore, gene expression of IL-36Ra was found to be significantly higher in these patients when compared with those suffering from Crohn's disease and non-inflammatory control groups [187]. Preclinical studies from IBD patient samples and animal models substantially support the IL-36 pathway's pro-fibrotic role in the gut; nevertheless, the search findings reveal no current status or updates on clinical trials specifically targeting this cytokine for the treatment of intestinal fibrosis. This might be a growing area of research and medication development, although specific clinical trials may still be required to assess the effectiveness and safety of IL-36/IL-36R inhibition for this particular purpose. Overall, it is critical to serve as a bridge between inflammation and fibrosis, to make it a potential therapeutic target for fibrotic disorders and performing preclinical and clinical research to assess the safety and efficacy of targeting IL-36 in diverse fibrotic disorders are all extremely important.

## Box 4 IL-36 contribute to the pathogenesis of autoimmune diseases

IL-36 cytokines all pertain to the IL-1 family and exhibit proinflammatory properties. Inappropriate IL-36 expression has been shown associated to an array of autoimmune ailments, including psoriasis, rheumatoid arthritis (RA), and primary Sjögren's. IL-36α is associated with skin abnormalities such as psoriasis and impetigo herpetiformis. IL-36β impacts on human synovial fibroblasts and articular chondrocytes, indicating a function for IL-36 in autoimmune disease inflammatory responses. *A471T* for IL36R, a single nucleotide gene polymorphism, leads to a substitution in the TIR domain that inhibits its interaction with IL-1RacP, and reduces IL-36R signaling.

## Prospects for the treatment of fibrosis

Fibrosis is principally dependent on the etiology of inflammation. While there are several potential targets for antifibrotic therapy, but currently no effective therapies. However, various small molecules or compounds are now being studied in clinical trials for fibrosis that have reported clinical data, as well as certain antifibrotic medications are currently within Phase 2, Phase 3 clinical trials, or are on the marketed. During the fibrosis process, a segment of the intestinal system is visibly decreased, affecting the basic function and structure of the intestinal tract and resulting in a significant loss in patient quality of life and prognosis. As a result, early detection, prevention, and treatment of chronic fibrosis and stenosis is critical to disease progression.

### Diagnosis

IBD-related chronic enteritis commonly results in intestinal fibrosis. Early detection of intestinal fibrosis is challenging, as well as stenosis. Fibrosis indicators such as fecal calcarine and C-reactive protein are used in diagnostic procedures. Non-invasive imaging techniques, such as barium contrast studies and cross-sectional imaging, as well as endoscopy and histology, can be beneficial in radiology. Current routine diagnostic tests are unable to distinguish between intestinal inflammatory and fibrotic alternations. It is also challenging to distinguish fibrosis from stenotic enteritis upon CT or MRI, since most stenoses exhibit both fibrosis. MRI was demonstrated to have reasonable accuracy in predicting the degree of inflammation in ileal CD and distinguishing substantial muscle hypertrophy from severe fibrosis with severe fibrosis, suggesting its diagnostic usefulness in the clinic [188, 189]. These, however, are insufficient for early clinical diagnosis. Bowel wall thickness as assessed by ultrasound is an effective marker of inflammation in small bowel CD, albeit relative enhancement by ultrasound or MRE does not distinguish between inflammatory activity and fibrosis [190]. Elastography assesses the degree of fibrosis and the extent of stenosis by ultrasound techniques [191]. According to glycoproteomics, liver growth factor-alpha and cartilage oligomeric matrix protein might be blood biomarkers for identifying and evaluating intestinal fibrosis [191]. Although CT enterography (CTE) has shown potential in the assessment of small intestinal disease, its accuracy in identify CD phenotypes remains unclear. Nonetheless, CTE is valuable in distinguishing between inflammatory and fibro-stenotic lesions. The study of specific CTEs and associated phenotypes, as well as PET/MR enterography, still needs to be improved in the future [192, 193]. The hallmark biochemical signs of fibrosis have yet to emerge. The best diagnostic techniques for detecting and assessing the degree of fibrosis in IBD patients in the early stages of disease activity, in particular, are not yet available.

### Prospective impact of cytokines on the fibrosis

There is currently no therapeutic way to totally prevent or reverse fibrosis. However, there are a few options for therapy for persons with IBD who develop intestinal fibrosis. Corticosteroids were administered to alleviate symptoms in a trial of 26 people with celiac disease and acute intestinal obstruction [194]. To assess the efficacy of anti-TNF medication in stenosing CD, researchers revealed that approximately two-thirds of the 97 patients treated with adalimumab had successful responses, with nearly half of the replies remaining until the conclusion of the follow-up period. Four years after beginning therapy, more than half of the patients had no undergone surgery [195]. Upon plainly ineffective in fibrosis and even stricture of the anti-inflammatory pharmaceutical therapy, endoscopic therapy, gastrointestinal stricture plasty, or gut resection are all options.

Endoscopic therapy for fibro-stenosing includes endoscopic balloon dilation (EBD), intraluminal corticosteroid or anti-TNF injections, and metallic biodegradable or detachable stents are also treatments for IBD [38]. Endoscopic dilation was successful in 90% of 1463 celiac disease patients in 32 retrospective studies. Clinical cure was achieved in 83% of patients, with short-term cure of complications [38], and endoscopic stents were effectively in another 2.7% of patients for other strictures, such as esophageal or colonic cancers [196]. Treatment in this manner has also been extensively researched in CD. Unfortunately, two-thirds of patients experienced serious complications, such as stent migration and fistula formation leading to perforation [197]. Despite the fact that they offer an advantage in avoiding stenosis, interchangeable and metal-removable stents, which are becoming popular methods to prevent fibrotic stenosis, are linked with a higher overall risk of problems [198, 199]. Thereby, clinical research and development of endoscopic therapy for the prevention of fibrotic stenosis remain a crucial.

On the one hand, present anti-inflammatory drugs cannot effectively prevent and relieve fibrosis because the possible hazards, such as increased risk of certain forms of cancer and allergic responses, exceed the benefits [38]. Endoscopy, on the other hand, has only a limited function in diagnosing the kind of stricture since fibrotic strictures now need endoscopic balloon dilatation or surgery [200]. As a result, surgical intervention for intestinal fibrosis and stricture is now standard practice [38]. Retrospective studies revealed those with CD who underwent surgery promptly after diagnosis had a lower risk of reintervention and less use of hormones and immunosuppressants [201], also lower the probability of clinical recurrence substantially [202]. In accordance with the above view, surgical resection and stenosis replacement can effectively treat stenosis with low risk, and low long-term recurrence rate. It is important to note that endoscopy and surgery is no substitute for managing intestinal fibrosis, and focused antifibrotic medicines require development.

## Limitation, prospects and conclusions

With the deepening of the research on intestinal fibrosis, our understanding of intestinal fibrosis and therapeutic coping strategies has improved. Intestinal fibrosis is intricate and governed by a plethora of inflammatory and non-inflammatory variables, including immunological response, intestinal microecology, environmental factors, genes, and oxidative imbalance, all of which interact to cause fibrosis. The heterogeneity in the pattern of inflammation and fibrosis within a stricture might cause biopsies to be sampled incorrectly, affecting the accuracy of diagnosis and therapy, yet this usually occurs in large patient cohorts. Despite the fact that people have recognized the pathogenesis and serious consequences of intestinal fibrosis and have made continuous breakthroughs in the field of the pathogenic mechanism of fibrosis in various organs, its underlying mechanism is still weak, particularly at the cellular, molecular, and even genetic levels, and there is a wide range of research space. Simultaneously, antifibrotic therapy remains a challenge. The prevalent wisdom that intestinal fibrosis is an unavoidable and irreversible ailment is progressively shifting as we get a better knowledge of the cellular and molecular processes underlying its etiology. Furthermore, research on imaging criteria and biomarkers of intestinal fibrosis is essential for an early diagnosis of intestinal fibrosis. Also, with the continuous development of high-throughput sequencing and other genetic technologies, the number and type of IBD-related gene loci have been continuously expanded, which is conducive to linking fibrosis with specific cells or genes and the disease and promoting the development of targeted therapies. More effective and innovative anti-inflammatory and anti-fibrotic medications will emerge as our understanding of fibrosis mechanisms improves.

## Data Availability

Not applicable.

## Abbreviations

CT: CT enterography
CD: Crohn's disease
DAMPs: Damage-associated molecular patterns
EBD: Endoscopic balloon dilation
EMT: Epithelial–mesenchymal transition
EndoMT: Endothelial-to-mesenchymal transition
F/B: Firmicutes/Bacteroidetes ratio
GM-CS: Granulocyte–macrophage colony-stimulating factor
IL: Interleukin
IBD: Inflammatory bowel disease
IRAKs: Interleukin-1 receptor-associated kinases
LPMCs: Lamina propria monocytes
LPS: Lipopolysaccharides
Mφ: Macrophage
M-CSFR-1: Macrophage colony-stimulating factor receptor-1
MAPK: Mitogen-activated protein kinase
MMP: Matrix metalloproteinases
NLRs: Nod-like receptors
NF-κB: Nuclear factor-κB
PHLFs: Primary human lung fibroblasts
PRRs: Pattern recognition receptors
PSMad2-3: Phosphorylated Smad2-Smad3
TGF-β: Transforming growth factor beta
TLRs: Toll-like receptors
TJP: Tight junction protein
TNF: Tumor necrosis factor
RA: Rheumatoid arthritis
RAAS: Renin–angiotensin–aldosterone system
ROS: Reactive oxygen species
RNS: Reactive nitrogen species
SHRs: Spontaneously hypertensive rats
SLE: Systemic lupus erythematosus
Th: T helper
TIMP: Tissue inhibitors metalloproteinases
TNF: Tumor necrosis factor
TL1A: TNF-like ligand 1A
UC: Ulcerated colitis
WT: Wild-type
